# Microbial control over carbon cycling in soil

**DOI:** 10.3389/fmicb.2012.00348

**Published:** 2012-09-26

**Authors:** Joshua P. Schimel, Sean M. Schaeffer

**Affiliations:** Department of Ecology, Evolution and Marine Biology, University of California at Santa BarbaraSanta Barbara, CA, USA

**Keywords:** microbial communities, carbon, diversity, litter, roots, soil

## Abstract

A major thrust of terrestrial microbial ecology is focused on understanding when and how the composition of the microbial community affects the functioning of biogeochemical processes at the ecosystem scale (meters-to-kilometers and days-to-years). While research has demonstrated these linkages for physiologically and phylogenetically “narrow” processes such as trace gas emissions and nitrification, there is less conclusive evidence that microbial community composition influences the “broad” processes of decomposition and organic matter (OM) turnover in soil. In this paper, we consider how soil microbial community structure influences C cycling. We consider the phylogenetic level at which microbes form meaningful guilds, based on overall life history strategies, and suggest that these are associated with deep evolutionary divergences, while much of the species-level diversity probably reflects functional redundancy. We then consider under what conditions it is possible for differences among microbes to affect process dynamics, and argue that while microbial community structure may be important in the rate of OM breakdown in the rhizosphere and in detritus, it is likely not important in the mineral soil. In mineral soil, physical access to occluded or sorbed substrates is the rate-limiting process. Microbial community influences on OM turnover in mineral soils are based on how organisms allocate the C they take up – not only do the fates of the molecules differ, but they can affect the soil system differently as well. For example, extracellular enzymes and extracellular polysaccharides can be key controls on soil structure and function. How microbes allocate C may also be particularly important for understanding the long-term fate of C in soil – is it sequestered or not?

Interest in how the composition of soil microbial communities governs the functioning of soil and ecosystem processes goes back to the dawn of microbiology with workers such as Pasteur and Winogradsky. However, the way we think about such issues has evolved – the focus is no longer identifying organisms with a capacity to carry out a function, e.g., characterizing the traits of specific nitrifiers (Waksman, 1927; Meyer, 1993) but on how varying the composition of a group affects the dynamics of the process it carries out – for example, how differences in the nitrifiers present affect nitrification kinetics (Braker and Conrad, 2011). This shift in the nature of the questions has been driven by three factors. The first is the development of techniques that allow us to characterize the identities of microbes *in situ* (16S and 18S rDNA), their potential (functional genes), and their physiological state (e.g., RNA/DNA ratios; stable isotope probing; BrDU incorporation; Roux-Michollet et al., 2010; Morales and Holben, 2011). The second is the growing interest in integrating evolutionary and ecological theory into microbial ecology to better understand microbial systems (Jiang, 2007; Prosser et al., 2007; Peay et al., 2008; Fierer et al., 2009; Locey, 2010). The third is the societal and scientific need to better understand and model important processes that influence ecosystem functions and the global climate system (Schimel and Gulledge, 1998; Reid, 2011).

In some ways, the thinking in microbial ecology has paralleled the development of plant ecology – questions have gone from “who’s there?,” analogous to the work of Joseph Banks and other naturalists in the late eighteenth and nineteenth centuries, to “why are they there?,” and “what are they doing?,” analogous to Tansley and Clements in the early 20th centuries. A question central to much of modern microbial ecology is “does who’s there matter?”

As microbial ecologists, we might wish the answer to this question to be “yes,” but in fact, this is not certain (Prosser, 2012). It is likely that for some processes the composition of the community matters, while for others it does not, and that the answer changes with physical and phylogenetic scale. Schimel (1995) postulated that “At a small enough scale, microbial community structure *must* be a dominant control on ecological processes, but as we move up in scale toward the ecosystem and integrate across many individual communities, the influence of individual community structures decreases.” That paper posed the question “Is there some minimal scale necessary to adequately explain ecosystem processes at which microbial community structure still has a measurable influence on the nature and rates of those processes?”

From that question grew the argument that “narrow” processes – those that involve a specific physiological pathway or which are carried out by a phylogenetically constrained group of organisms – might be sensitive to the composition of the guild of microorganisms carrying it out, even at the ecosystem level (Schimel, 1995; Groffman and Bohlen, 1999). Examples of such processes include specific plant–microbe interactions (e.g., N-fixation, mycorrhizae, and pathogens) and trace gas emissions, notably N_2_O and CH_4_. Both the conceptual arguments and the research to back them up are increasingly well developed for such processes. For example, the ratio of N_2_O/N_2_ produced by denitrification (Bakken et al., 2012; Salles et al., 2012), the rate of nitrification and the speed with which it responds to fertilization (Isobe et al., 2011), and the sensitivity of methanogenesis to ${NH}_{4}^{+}$(Bodelier et al., 2000) are all sensitive to the composition of the community of organisms carrying them out.

However, these narrow processes are generally niche players in overall biogeochemical cycles. They are often important, either for ecosystem functioning (e.g., nitrification or sulfate reduction) or for global systems (e.g., N_2_O and CH_4_ fluxes), but typically engage only a small fraction of the total microbial community and are responsible for a limited portion of the total cycle of the involved element. Most microorganisms in soil are aerobic heterotrophs involved in the “broad” or “aggregate” processes (*sensu*;**Schimel et al., 2005); these are the processes that are carried out by a wide range of organisms or that we measure as a single process but are actually the sum of multiple distinct processes (e.g., soil respiration). Broad processes are responsible for the largest flows of C in soil systems: decomposition and C storage.

In this paper, therefore, we will focus on the microbial role in these large flows associated with the soil C cycle. We will briefly discuss our evolving understanding of the nature and causes of microbial diversity in soil to consider the level of phylogeny that might define meaningful functional groups for addressing “who’s there” questions in C-cycling research. We will then discuss the circumstances where microbial community structure might regulate the processing of organic matter (OM) in soil, and some areas where we see a particular need for advancing this research.

## CAUSES AND NATURE OF MICROBIAL DIVERSITY IN SOIL

Microbial diversity in soil is high. Typical soil samples contain many thousands of individual taxa (commonly described as “operational taxonomic units”; OTU’s) of Bacteria, Archaea, and Fungi. Some estimates suggest there can be more than 10^6^ individual species-level OTUs in a single soil (Fierer et al., 2007). This poses two central, but related, questions to microbial ecologists: how there can be such great diversity, and does it have any functional significance? (Prosser, 2012).

Classical theories of biodiversity are grounded in the concept of the niche and competitive exclusion: two species cannot stably coexist in a single niche. Thus, each species *must* have some functional differentiation (Clark, 2010). Niche-based theory, however, struggles with the high biodiversity of plants, which compete for a limited suite of resources (light, water, mineral nutrients); yet there may be hundreds of species within some habitats.

To explain such anomalous patterns of high biodiversity, alternative ideas have developed. Neutral theory argues that species can coexist within a niche when the variation in fitness among individuals is as great as among species (Hubbell, 2001); but species can also coexist when competition among individuals is as intense as among species (Clark, 2010; Clark et al., 2011; Beckage et al., 2012). Such dynamics allow functionally overlapping taxa to coexist, especially when the environment is highly variable, and when organisms are sessile; conditions that likely hold true for many soil microbes (Sloan et al., 2006; Dumbrell et al., 2010; Fierer and Lennon, 2011; Prosser, 2012).

Growing evidence suggests that niche and functional differentiation explain patterns of diversity at high levels of microbial phylogeny (e.g., families and phyla) and is associated with life-history strategies (Fierer et al., 2007; Philippot et al., 2010). However, it has been hard to identify meaningful functional differentiation within more finely defined groups (e.g., within genera; Philippot et al., 2010; Prosser, 2012). This conclusion, if true, will affect how we study microbial community composition; for example, how deeply to sequence communities to analyze their structure in terms of ecologically meaningful groups. It takes far fewer sequences to quantify a community to family than to species (Barberán et al., 2012).

In soil, organisms must adapt to a complex array of substrates, physical/chemical conditions, and biotic interactions, each of which may affect community composition. Some organisms specialize on particular substrates; for example, fungi that grow best on sucrose vs. cellulose vs. lignin vs. tannin–protein complexes (Hanson et al., 2008). In other cases, organisms appear to respond to specific environmental variables. For example, O_2_ (Bodegom et al., 2001), moisture (Lennon et al., 2012), pH (Fierer and Jackson, 2006) and even varying levels of these parameters (DeAngelis et al., 2010) can select for specific organisms. In some cases, this selection operates at high phylogenetic levels – e.g., pH controls the relative growth of fungi vs. bacteria (Rousk et al., 2009). In other cases, selection operates at family or genus. For example, within the phylum Glomeromycota (the arbuscular mycorrhizal fungi), soil pH may select more strongly than host plant for the specific taxa present (Dumbrell et al., 2010); within the bacteria, *Clostridium *spp. are obligate anaerobes while most *Bacillus *spp*.* are aerobes, yet both genera are closely aligned within the Firmicutes.

However, the dominant environments that we can identify in soil are rarely defined by single characteristics (e.g., pH alone), but by combinations of characteristics that organisms must deal with in synchrony. To adapt to an environment with a suite of co-occurring conditions, an organism requires a suite of complementary traits – a life history strategy. For example, litter decomposers (e.g., many basidiomycete fungi) rely on extracellular enzymes to cut plant polymers into oligomers and monomers that may be taken up and metabolized, but they must also deal with litter drying out frequently (Schimel et al., 1999) and with the high C/N stoichiometry typical of leaf litter. Some bacteria are “rhizobacteria” that appear to depend on specific exudates released by plant roots (DeAngelis et al., 2009; Remenant et al., 2009) and are adapted to the complex environment of the rhizosphere (Bertin et al., 2003). The Acidobacteria appear to be stress tolerant oligotrophs (Fierer et al., 2007, 2011) while the Bacteroidetes and the β-Proteobacteria appear to be copiotrophs that require adequate moisture (Lennon et al., 2012).

Evolving a successful life history strategy implies deep evolutionary patterns and may explain why we observe meaningful functional groups or guilds of microbes, and that they are defined primarily at high phylogenetic levels – families or phyla rather than at species or genera (Moorhead and Sinsabaugh, 2006; Fierer et al., 2007; Philippot et al., 2010; Follows and Dutkiewicz, 2011). For example, the ability to retain high levels of rRNA through drought and to respond quickly to rewetting appears to be a function of bacterial phylum, with Actinobacteria and Verrucomicrobia being rapid responders, while Firmicutes were intermediate; Proteobacterial responses however differed at the class level (Placella et al., 2012). At the species level, there generally appears to be substantial functional redundancy (Prosser, 2012).

This conclusion is reinforced by the ubiquity of horizontal gene transfer (HGT) among bacteria. HGT allows organisms to transfer the genes to carry out specific processes (Kurland et al., 2003). However, HGT is most common among closely related taxa (Kurland et al., 2003), and for pathways that are simple and require few enzymes. Thus, HGT is unlikely to break down functional barriers at high levels of phylogenetic difference and so is unlikely to transfer major life-history strategies that require complex gene networks or rearranging core physiological pathways (Kurland et al., 2003). For example, while the genes for nitrogenase appears to have been transferred across taxa multiple times (Falkowski et al., 2008), transferring the full suite of genes required to form N-fixing nodules in legumes appears to have happened perhaps twice in history (Chen et al., 2003). Denitrification has spread widely across the bacterial world because it requires only a slight modification to the terminal end of the electron transport chain, branching electrons off from cytochrome *b* to one of several nitrogen reductases. It is an easy physiology to maintain as an alternate to aerobic respiration. In contrast, because the redox potential for sulfate reduction is so much higher than that of O_2_, organisms cannot merely insert sulfate reductase in place of cytochrome *o*; sulfate reducers have an entirely different electron transport system (Rabus et al., 2006), making it difficult for an aerobe to become a ${so}_{2}^{=}$reducer through HGT. The ability to carry out specific biodegradation reactions has been transferred frequently (Liang et al., 2012), but a rhizobacterium is not likely to become a litter decomposer overnight.

Much soil diversity may actually reflect beta diversity – a diversity of habitats within a landscape, rather than diversity within a habitat. This argues that soil is really a complex landscape with repeatable and definable microhabitats, each of which might have more constrained diversity. No one considers it surprising that California has > 3,000 native plant species because the State spans from alpine tundra to conifer forests to arid scrubland and desert, each with its own array of species. Does soil have analogous distinct communities? Rhizospheres select for discrete and reproducible communities, based on both the chemical nature of plant rhizodeposits (Paterson et al., 2007; DeAngelis et al., 2009; Dennis et al., 2010) and the physical environment created by roots (e.g., altered O_2_, pH, and water availability; da Rocha et al., 2009; Hinsinger et al., 2009). Soil aggregates may also select for specific microbial groups; for example, Acidobacteria may be common in macroaggregates but not the inner microaggregate (Mummey et al., 2006). Communities may also vary based on the size pores they inhabit (Ruamps et al., 2011).

However, even if physical structure does not create repeated defined habitats that select for specific communities analogous to grassland or alpine plant communities, the physical complexity of the microbial landscape and a lack of connectivity between pores may reduce competitive interactions among taxa and allow greater overall diversity (Görres et al., 1999; Dechesne et al., 2008) analogously to how different valleys within a single mountain range may have somewhat different flora. Within a single soil, decreasing pore connectivity by reducing water content can increase bacterial species richness (Carson et al., 2010). We still only poorly understand how soil structure creates habitats and niches and how it regulates interactions to control diversity and community composition. This remains an important research area (Schmidt et al., 2011; Dungait et al., 2012).

Questions remain, however, about how many distinct niches can exist in soil, and about the level of phylogenetic resolution at which meaningful niche selection and partitioning acts. As we explore the functional significance of community composition, we need to further develop our understanding of the nature of microbial diversity and the phylogenetic levels at which distinct life-history strategies emerge and how these translate into meaningful microbial functional groups and thence into meaningful functions. We also need to better understand the “microbial landscape” and how the physical structuring of the soil system interacts with microbial communities to regulate the processes that control ecosystem functioning.

## CONSEQUENCES OF SOIL COMMUNITY COMPOSITION

As different groups of microorganisms have distinct functional traits with the potential to influence the processes they carry out (e.g., exoenzyme producers vs. “cheaters”; Allison, 2005), it raises the question of where and how those differences might be expressed in the environment. Just because organisms’ traits differ does not mean that they necessarily function differently. There are several necessary conditions for soil microbial community composition to affect ecological processes.

1. Organisms must differ in their functional traits.
2. Biological reactions must be either:
  a. The rate limiting step in a reaction sequence or,
  b. The fate-controlling step – i.e., at a branch point that channels substrates into pathways with different fates.

The first criterion is the basis of the concept of “physiologically broad” within “broad vs. narrow” theory: if all organisms carry out a process in the same way it can not matter which is active for process function. Community composition can only affect processes if organisms vary in how or when they function (Schimel, 1995; Allison and Martiny, 2008).

Even if the organisms present in a community do vary in their functional characteristics, being different is not enough to control the dynamics of C cycling. The organismal biology must also be what controls the process – either in terms of how fast a reaction proceeds or where it proceeds to: what are the products.

### RATE LIMITING STEP: CATABOLISM VS. ACCESS?

**FIGURE 1:**
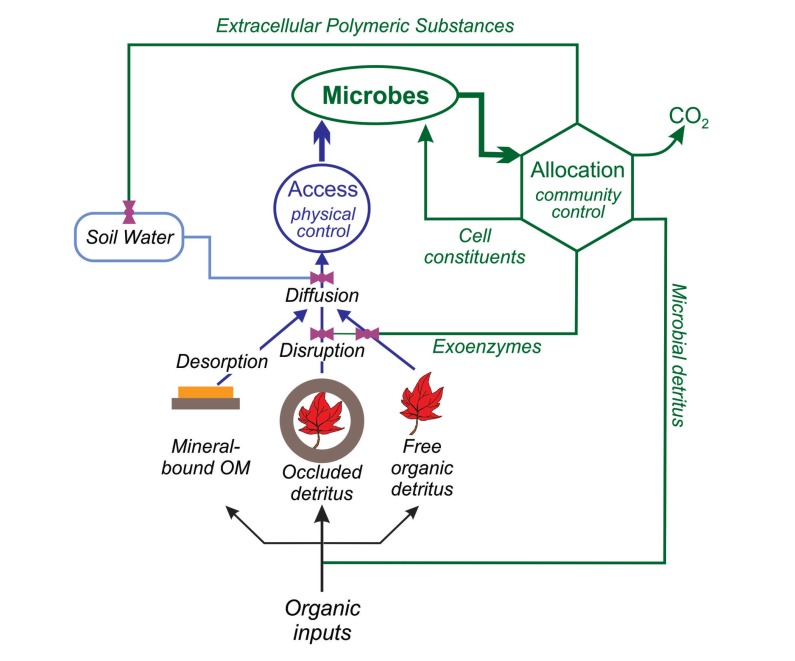
**Microbial C cycling: the relative roles of physical access to soil C pools and of microbial allocation patterns in regulating overall soil C dynamics**.

Most OM transformations involve multiple steps with different inherent kinetics. For microbial community composition to play a role in controlling such a transformation, the slowest, rate limiting step, must be biological. In soil, that is not necessarily the case; rather abiotic processes can be rate-limiting (Kemmitt et al., 2008). For example, in dry and sunny ecosystems, photodegradation of aboveground litter can potentially oxidize plant-C all the way to CO_2_ (Austin and Vivanco, 2006). Photodegradation, however, generally accounts for only a limited amount of litter breakdown (Brandt et al., 2010). The more common way for abiotic processes to regulate OM turnover is through physical mechanisms that limit microbial access to substrate (Stevenson, 1982; Pingnatello, 1999; Kemmitt et al., 2008; Dungait et al., 2012). Thus, in considering the potential role of microbial community composition in a biogeochemical process, the first question is whether microbes have physical access to the substrate (**Figure 1**).

In fresh unprotected detritus, microbial access is not generally a constraint on decomposition. The exception is wood; it can take some time for fungal hyphae to penetrate into a log (Barker, 2008). In mineral soils, however, the situation is different. Mineral soils contain the bulk of OM in the total soil profile, and possibly in an entire ecosystem (Jobbágy and Jackson, 2000). Much of this is in protected forms, either occluded in aggregates or sorbed on mineral surfaces (**Figure 1**; Krull et al., 2003). The rate at which this C can be metabolized is limited by microbes’ ability to access it (Six et al., 2004; John et al., 2005). The importance of physical protection has long been recognized as a control on soil OM (SOM) turnover (Yoo et al., 2011), but increasingly researchers have been recognizing the role of physical space and the structure of the “microbial landscape” as a specific control on the dynamics of microbial communities and of their function as well (Dungait et al., 2012).

Microbes in mineral soils are constrained by the pore networks that they live in (Young and Ritz, 2005; Donnell et al., 2007). As soils dry, bacteria become effectively immobile (Wang and Or, 2010) and must rely on diffusion to supply resources. Yet, soil is a “sticky” environment, and substrate diffusion can be slowed or prevented when molecules interact with electrically charged clay particles or OM that coats particles (Carrington et al., 2012). In other words, life in soil is like life in a chromatography column. The interactions of microbes and substrates with the physical matrix regulates how, or even whether, soil C is utilized; the critical processes are sorption/desorption, diffusion, and transport (**Figure 1**; Ekschmitt et al., 2005).

For example, in a California grassland soil, Xiang et al. (2008) showed that in deep soils (1 meter), multiple dry/wet cycles increased total respiration and microbial biomass by more than 500%; the C respired came from a pool with an estimated turnover time of 600–800 years (Schimel et al., 2011). This pool of chemically labile OM was only metabolized following rewetting either because rewetting caused it to desorb, or because the flow of water redistributed C and so overcame diffusion limitations.

A meta-analysis of studies evaluating microbial respiration responses to water stress (Manzoni et al., 2012) showed that across a wide range of soils, the relationship between water potential and relative respiration is linear with a consistent threshold value at which respiration effectively stops (ca. -14 MPa); this value was similar to “the water potentials where soil diffusion becomes impaired.” They concluded that the only mechanism that could reasonably explain the observed relationship was diffusion control on substrate supply.

In contrast, when aggregates occlude organic detritus (Six et al., 2004), two steps are required for microbes to process the material – first a physical step of aggregate disruption, and then possibly a second step in which exoenzymes break up the polymers (Navarro-García et al., 2011). The aggregate disruption step still limits overall metabolism of the material, but once that step is overcome, there may still be a delay before material is processed and metabolized.

The conclusion that OM breakdown in mineral soils is not limited by the catabolic capacity of the OM, but rather physical factors that limit microbial access to it, is reinforced by our changing understanding of the chemical structure of SOM. The classical model of SOM was based on humic/fulvic acids, and what might be described as the “snowflake” theory of SOM, in which humic materials were thought to be large polymers (Sutton and Sposito, 2005), molecules so complex it was possible no two were identical. In such a model, molecules require extracellular enzymes to fragment them, but with no repeated structures, enzymes could not be high affinity “latch-and-key” hydrolytic enzymes but instead must be non-specific oxidative “shotgun” enzymes that produce high-energy radicals or peroxides. Thus, microbes would rely on chance to generate fragments that they can take up and metabolize (Stevenson, 1982). In this vision, SOM molecules are inherently resistant and dependent on specialized exoenzymes; decomposition is therefore potentially sensitive to the organisms involved, and whether they have the necessary traits to be able to process humic molecules.

The old humic model of SOM, however, is increasingly considered obsolete (Schmidt et al., 2011), and is being replaced by a conceptual model in which SOM is made up of aggregates of small, but chemically defined molecules (Sutton and Sposito, 2005; Schmidt et al., 2011). These small molecules can also form layers on clay surfaces, leading to a model that has been called the “onion layering” model (Sollins et al., 2006). In this new model, individual constituent molecules are small enough that they can be taken up by microorganisms and are often simple enough that they can be channeled into metabolic pathways that are common among microbes. There should therefore be a lot of redundancy among microbes’ ability to use such compounds, making it unlikely that their metabolism would be limited by a lack of appropriate enzymes. Yet, the bulk mineral-phase carbon in soils is frequently thousands of years old (Trumbore, 2009). Because such long turnover times cannot be explained by chemical recalcitrance (Dungait et al., 2012), they are unlikely to result from biological constraints and are presumably insensitive to microbial community composition.

### FATE CONTROLLING STEP: ALLOCATION

Although we argue that for the protected OM in mineral soils, the composition of the microbial community is not important in controlling the *rate* at which SOM is processed, that does not mean community composition is not important in the *fate* of this material. While a molecule’s accessibility to a microbe is controlled by physical processes, once a microbe has taken it up, its fate is entirely under the control of that organism (**Figure 1**). Ultimately therefore, while catabolic potentials have limited role in controlling the turnover of mineral SOM, anabolic processes are unquestionably important (Liang et al., 2011). What do microbes do with the carbon they access? How do they allocate it? How do microbial C transformations further affect the composition of SOM? These *are *sensitive to microbial community structure.

The sensitivity of SOM turnover to anabolism is recognized implicitly even in large-scale biogeochemical models; although these models often lack any microbial carbon pool, and even when such a pool exists it is usually just a C reservoir, rather than a driver of decomposition (Manzoni and Porporato, 2009). In these models, when C moves from one pool to another, some is lost as CO_2_; the proportion of C-moved relative to C-lost is essentially equivalent to carbon use efficiency (CUE). In physiology, CUE is the fraction of substrate that is taken up that is assimilated immediately into microbial biomass (Allison et al., 2010). With simple compounds, the immediate CUE – usually measured by a short-term assay with an isotopically labeled substrate – is more a function of molecular structure than microbial community composition (Sugai and Schimel, 1993). The magnitude of CUE, however, is a function of time and physiological condition; stressed microbes can have a higher maintenance energy demand which functionally reduces CUE (Allison et al., 2010). Thus, how CUE and C-turnover respond to stress may be a function of microbial life history strategy and stress tolerance.

Microbes, however, do more with substrate than just convert it to “biomass.” Rather, they synthesize a variety of products that affect the functioning of ecosystems. A select few of these include:

A. Extracellular enzymes
B. Extracellular polysaccharides
C. Cell wall polymers: amino sugar-based peptidoglycan and chitin
D. Stress response compounds: osmolytes, cryoprotectants, chaperones etc. (Schimel et al., 2007).

The fate of each group of compounds is different in soil, and patterns of allocation and production vary across microorganisms (Harris, 1981; Lennon et al., 2012). For example, the microbial products present in decomposing aspen litter differed dramatically with the site the litter was decomposed in (i.e., microbial inoculum), even though decomposition rates did not differ significantly (Wallenstein et al., 2010). Substantial differences among allocation patterns are associated with microbes’ life-history strategies and hence with their phylogeny. Further, the ways in which microbes allocate C can influence soil structure and function and so alter microbial habitats and overall soil functioning.

#### Extracellular enzymes

For those organisms that rely on detritus (plant, animal, or microbial) for their resource supply, extracellular enzymes are required to break down polymers (Sinsabaugh, 1994). Although several simple theoretical models of decomposition include a single enzyme pool (e.g., Schimel and Weintraub, 2003), in reality a suite of different enzymes are required, including substrate-specific enzymes targeting C, N, and P (e.g., cellulolytic, proteolytic, and phosphatase), and non-specific oxidative enzymes such as laccase and peroxidase (Caldwell, 2005; Sinsabaugh et al., 2009). For exoenzyme-producing decomposers, enzymes may be the first priority for C-allocation to ensure resource supply (Schimel and Weintraub, 2003). The overall microbial community is able to shift allocation among these different groups of enzymes to match production to resource demand; enzymes are selectively produced to increase the supply of the most limiting element (Sinsabaugh and Moorhead, 1994; Sinsabaugh et al., 2009) and to target the most available substrates (Sistla and Schimel, 2012). It remains unclear how much of this is due to physiological plasticity of individual organisms or reflects shifts in the composition of the microbial community. The products of exoenzyme breakdown become available to other organisms including other microbes, so called “cheaters” (Allison, 2005) or “opportunists” (Moorhead and Sinsabaugh, 2006), and to plant roots (Schimel and Bennett, 2004). Some microbial groups are dominant producers (e.g., Basidiomycetes) while cheaters appear to dominate in groups such as the β-Proteobacteria and Bacteroidetes (Fierer et al., 2007).

#### Extracellular polysaccharides

Another important class of extracellular materials is polysaccharides (Holden, 2011; Sutherland, 2001). Some microorganisms can embed themselves in a matrix of extracellular polymeric materials (EPS) that are mostly polysaccharide but also contain DNA and protein (Or et al., 2007; Jiao et al., 2010; Holden, 2011). EPS is hygroscopic (Chenu, 1993; Henao and Mazeau, 2009), facilitating prolonged cellular hydration and nutrient resupply in drying soils (Or et al., 2007). EPS can bridge between microbes and their substrates and allow them to survive in dry soils. EPS can also protect exoenzymes (Sutherland, 2001; Holden, 2011), and can either promote (Chenu and Roberson, 1996) or constrain (Holden et al., 1997) C diffusion to microbes. EPS can alter soil structure to mediate water retention (Chenu and Roberson, 1996), hydraulic conductivity (Henao and Mazeau, 2009; Rosenzweig et al., 2009), and aggregate structure (Roberson et al., 1995; Park et al., 2007). By promoting aggregate formation, microbes can create favorable growth environments, either in the interior of macroaggregates, which may have increased water content, proximity to substrates, and physical protection from predators (Görres et al., 1999; Neher et al., 1999; Six et al., 2006), or in macropores that offer easier access to diffusing substrate (Ruamps et al., 2011). Aggregate interiors, however, can also be a constrained growth environment when accessible substrates are depleted, or if intra-aggregate pore size is small enough to prevent colonization (Chenu et al., 2001).

Microorganisms’ ability to produce EPS appears correlated with their ability to grow at low water potentials (Lennon et al., 2012) and so appears to be part of a deeply rooted life-history strategy that includes the ability to tolerate low O_2_ (associated with being in a saturated biofilm) and a longer lag before starting exponential growth. This strategy is concentrated in a subset of phyla, notably the Firmicutes (Lennon et al., 2012). Microorganisms can also produce other chemicals that directly affect soil conditions and structure, including a variety of proteins such as hydrophobins, glomalin (produced by arbuscular mycorrhizal fungi in the Glomales), and chaplins (produced by Actinomycetes; Rillig et al., 2007).

#### Cell wall polymers

Within a cell, microbes also have the ability to shift resource allocation among different pools; these allocation patterns affect the functioning of the cell itself and potentially that of the overall soil system. Cell wall materials are thought to be potentially important sources of C and N for long-term stabilization (Liang et al., 2011). The proportion of chitin, peptidoglycan, lipids, and other cell-wall and outer-membrane components depend on the ratio of Fungi:Bacteria:Archaea, as well as the proportion of Gram-positive:Gram-negative bacteria within the bacterial community. Amino sugars, which both bacteria and fungi use as components of their cell walls (peptidoglycan and chitin, respectively) become important constituents in soil organic nitrogen pools (Dai et al., 2002; kögel-Knabner, 2002; Joergensen and Wichern, 2008), yet are produced differentially by the different groups of organisms.

#### Stress response compounds

It has been argued that microorganisms physiologically acclimate to survive stresses such as low water potential or freezing by accumulating cytoplasmic constituents such as osmolytes or cryoprotectants (Schimel et al., 2007). These molecules would have to be lost or transformed rapidly when the stress ended (rewetting or thaw). While there are consistently flushes of C and N upon rewetting and thaw, recent research suggests that in soil the “osmolyte theory” for how microbes tolerate drought may be incorrect (Boot et al., 2012; Kakumanu et al., 2012) and that the substrate flushes may be instead be associated with mobilization of non-biomass SOM (Miller et al., 2005; Xiang et al., 2008). This may not be the case for freezing, however, as the organisms that are active at low temperature appear distinct from those active under warmer conditions (McMahon et al., 2011) and the nature of OM processing and nutrient balance shifts between summer and winter (Schimel and Mikan, 2005). Soil freezing may select for anaerobes (Miller et al., 2007). How the production of such material varies across the microbial world remains unclear.

## SCALING UP IN TIME: DO MICROBIAL COMMUNITY INFLUENCES SHIFT WITH TIME SCALE?

The importance of microbial community composition may vary with the time scale being considered, but not necessarily in an intuitive way. Generally, we assume that scales of time and space are linked – it makes no sense to think about the global carbon cycle *this second*, nor does it make sense to ask what carbon in a single square centimeter will be doing over the next century. Rather we assume that questions framed at fine spatial scales will also target short time scales – studies on soil cores rarely last longer than months. In considering spatial scales, Schimel (1995) suggested that there should be a continuum in the influence of microbial community structure, with maximal influence at the finest spatial scales, with influence fading out at progressively larger spatial scales. We suggest that this is reasonable, but the same logic might not apply to time scales.

As the scale of focus changes, the nature of the processes that are most relevant change, as do the intellectual models that we use for considering them. For example at the pore-scale, we focus on patterns of microbial growth and substrate diffusion; reaction-diffusion models are a core intellectual paradigm for understanding this scale (Or et al., 2007; Holden, 2011); these emphasize access and catabolism. A new approach in microbial ecology is Dynamic Energy Budget modeling, which focuses on how organisms allocate resources under different environmental conditions and how that translates into population growth and turnover (Klanjscek et al., 2012). At the ecosystem scale, studies frequently focus on a seasonal to interannual time scale and emphasize plant production and decomposition (the life and death histories of plants) – box-and-flow biogeochemical models are the dominant paradigm (Manzoni and Porporato, 2009) and at this scale it is difficult to show any substantial influence of microbial community composition on C cycling. Larger scales become the domain of Earth System models, but these generally have a biogeochemistry core that is based on ecosystem-scale models (Clark et al., 2011). One might therefore argue that at these larger scales, the specific dynamics of microbes would be even less important in regulating the key processes. But at the decade-and-up times scales relevant to the global climate system, plant production and litter decomposition become tightly balanced, and overall C storage and loss become increasingly a function of the big, slow pools – e.g., stabilized OM in the mineral soil (Jobbágy and Jackson, 2000). Thus, to evaluate the processes that control C-sequestration, it is not the rate of microbial growth or of litter decay that matter most – it is the production of stabilized materials, which are a small fraction of the total (Liang et al., 2011). These products result from specific anabolic pathways of microbes and on patterns of microbial community composition that are reasonably stable across time (Grandy et al., 2009). Thus, as we focus on understanding soil C dynamics overlong time-scales, the allocation patterns of specific groups of microbes that regulate the fate of OM in mineral soils becomes increasingly important.

## SCALING UP IN SPACE: WHERE IN THE SOIL ARE COMMUNITY INFLUENCES LIKELY IMPORTANT?

**FIGURE 2:**
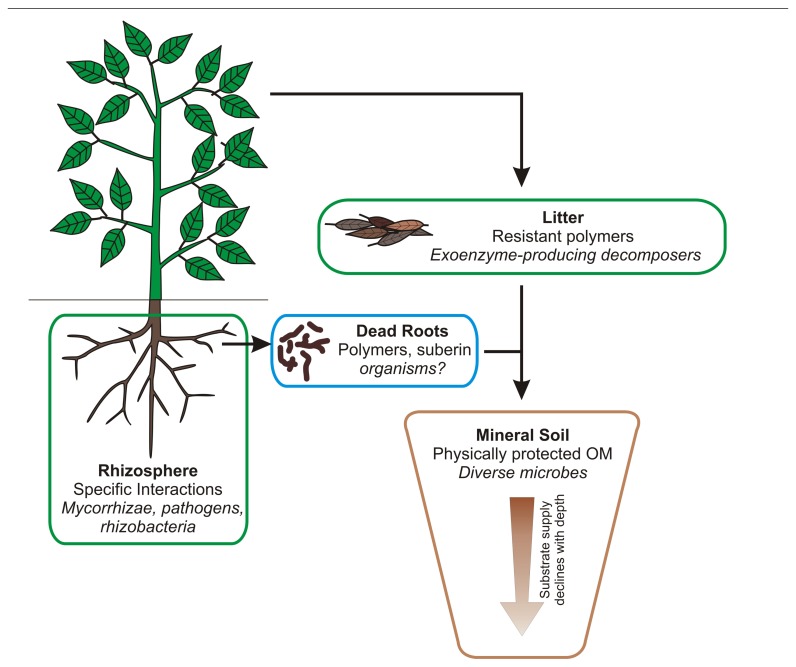
** The main zones in soil, the characteristics that regulate microbial functioning within each zone, and the dominant guilds of microbes present.** In litter and the rhizosphere (outlined in green), microbial community composition likely affects both the rate of processes and the fate of C. In mineral soil (outlined in brown), microbial community composition likely only controls the fate of C. In dead roots, community composition probably regulates both rate and fate, but little research has been done on this compartment.

We have briefly discussed perspectives on microbial diversity and the ways it may affect C cycling in soil systems. We have argued that our perspectives shift among time scales and specific processes, and that the roles of microbial community composition in controlling these processes shift as well. We also need to consider how these dynamics play out in different compartments of the soil system, because community influences may act differently among them. As a simple breakdown of the dominant zones within the soil landscape, we can consider (A) rhizosphere, (B) aboveground litter, (C) dead roots, and (D) mineral soil (**Figure 2**). We hypothesize that the composition of the microbial community plays a significant role in controlling C cycling in the rhizosphere and in organic detritus (litter or dead roots), but for distinctly different reasons. In the rhizosphere, microbial community composition is regulated by the specific substrates and chemical signals released by the plant root, and by the specific physical and biotic environment created by the plant root in terms of O_2_, pH, and other chemical variables (Jaeger et al., 1999; Hinsinger et al., 2009). These select for a distinct group of microbes, some of which act as plant growth-promoting rhizobacteria or as pathogens and so have powerful feedbacks to plant growth and C cycling. In physically unprotected organic detritus, chemical structures remain complex and specific to the plant, microbe, or animal that produced them; exoenzyme breakdown is necessary for microbes to metabolize them. Thus, their breakdown remains under biological control and sensitive to the specific identities of the decomposers present. Extensive work has been done on aboveground litter, linking chemistry and organisms, and decomposition dynamics. The community present in litter alters decomposition kinetics, often the community native to the litter’s home site is more effective than communities from other sites; the “home-field advantage” (Sinsabaugh and Moorhead, 1994; Schimel et al., 1999; Aneja et al., 2004; Craine et al., 2007; Strickland et al., 2009; Baumann et al., 2011; Freschet et al., 2011; Baldrian et al., 2012; Schneider et al., 2012).

In contrast, comparatively little research has been done on dead roots. This is surprising because typically at least half of ecosystem net primary productivity goes into belowground structures (Chapin III et al., 2002), and root litter is likely a greater source of stabilized soil C than is aboveground litter (Rasse et al., 2005; Schmidt et al., 2011; Carrington et al., 2012). The chemistry of root litter differs from that of foliar litter (Harmon et al., 2009), and differs in decomposition dynamics. For example, while high C/N foliar litter inevitably shows a phase of N-immobilization early in decomposition, this is not the case for roots, which begin to mineralize N in parallel with mass loss (Parton et al., 2007). Relatively few studies have evaluated the microbiology of root decomposition compared to those on leaf litter (e.g., Fisk et al., 2010; Baumann et al., 2011). Because root litter is such an important source of soil C and because so little has been done on the relationships between chemistry and microbiology in dead roots, we consider this to be the most important compartment for microbial study in the soil system.

In mineral soils, as we have discussed, the influence of microbial community composition on the rate of breakdown of SOM is likely to be limited. Rather, community composition may more strongly reflect the physical environment and substrate access patterns. However, the distribution of major phylogenetic groups may control the fate of that material. Here, we need continued study on SOM chemistry to better understand the factors that regulate microbial access to substrate and we need increased study on microbial processing and production of new materials to better understand how they regulate the physical structure of soil and the long-term fate of soil C.

## CONCLUSION

In their effects on soil C cycling, the influences of microbial communities appear to be associated with life history patterns that are deeply rooted in microbial phylogeny – functional groups appear at the level of families or phyla rather than species or genera. Even accepting that soil microbes have different life history strategies and comprise different functional groups however, for those differences to influence ecosystem C dynamics, those organisms must carry out steps in OM processing that are rate-limiting in overall OM breakdown, which requires that they have physical access to the material, or they must control the fate of that material, synthesizing alternative products with important characteristics for ecosystem function. The specific compounds produced likely affect the nature of soil processes most strongly at either the shortest or the longest time scales, but least strongly at the interannual “ecosystem” scale that dominates much biogeochemical study. In the rhizosphere and in detritus, community composition likely influences C-cycling rates, while in the mineral soil, it may primarily influence the fate of C, while physical processes controlling microbial access to C regulate turnover rate. The largest uncertainty about the role of community composition probably exists for dead roots. These may constitute the largest source of C that is sequestered in the soil and so represent the biggest long-term input yet is the least studied part of the system.

## Conflict of Interest Statement

The authors declare that the research was conducted in the absence of any commercial or financial relationships that could be construed as a potential conflict of interest.
